# Virtual Bystanders in a Language Lesson: Examining the Effect of Social Evaluation, Vicarious Experience, Cognitive Consistency and Praising on Students' Beliefs, Self-Efficacy and Anxiety in a Virtual Reality Environment

**DOI:** 10.1371/journal.pone.0125279

**Published:** 2015-04-17

**Authors:** Chao Qu, Yun Ling, Ingrid Heynderickx, Willem-Paul Brinkman

**Affiliations:** 1 Interactive Intelligence Group, Delft University of Technology, Delft, the Netherlands; 2 Human Technology Interaction Group, Eindhoven University of Technology, Eindhoven, the Netherlands; University of Udine, ITALY

## Abstract

Bystanders in a real world's social setting have the ability to influence people’s beliefs and behavior. This study examines whether this effect can be recreated in a virtual environment, by exposing people to virtual bystanders in a classroom setting. Participants (*n* = 26) first witnessed virtual students answering questions from an English teacher, after which they were also asked to answer questions from the teacher as part of a simulated training for spoken English. During the experiment the attitudes of the other virtual students in the classroom was manipulated; they could whisper either positive or negative remarks to each other when a virtual student was talking or when a participant was talking. The results show that the expressed attitude of virtual bystanders towards the participants affected their self-efficacy, and their avoidance behavior. Furthermore, the experience of witnessing bystanders commenting negatively on the performance of other students raised the participants’ heart rate when it was their turn to speak. Two-way interaction effects were also found on self-reported anxiety and self-efficacy. After witnessing bystanders’ positive attitude towards peer students, participants’ self-efficacy when answering questions received a boost when bystanders were also positive towards them, and a blow when bystanders reversed their attitude by being negative towards them. Still, inconsistency, instead of consistency, between the bystanders’ attitudes towards virtual peers and the participants was not found to result in a larger change in the participants’ beliefs. Finally the results also reveal that virtual flattering or destructive criticizing affected the participants’ beliefs not only about the virtual bystanders, but also about the neutral teacher. Together these findings show that virtual bystanders in a classroom can affect people’s beliefs, anxiety and behavior.

## Introduction

Human’s behavior, attitudes, emotions and cognition are extensively influenced by other people’s opinions. People establish beliefs about these opinions regularly through one-to-one conversations with another person. Often, however, other individuals are present during such a social interaction; for example, fellow students in a class when a student talks to a teacher, colleagues in a meeting when someone talks to his or her boss, or people in a queue overhearing a person talking with someone at an information desk. Even though, these individuals, the so called bystanders, do not directly participate in the conversation, they may whisper or use nonverbal cues to express their opinion about what is being said.

Research showed that humans may be affected by the behavior of surrounding bystanders [1] and that these bystanders may play an important role in human-human social interaction [2]. For example, behavior and judgments of a group of peers may influence an individual’s cognition and judgment [3, 4]. This behavior may include words, intonations, gestures and facial expressions [5]. Direct interaction with a virtual human [6–8] or virtual group [9–12] has received considerable research attention, but this research has largely ignored the role of bystanders, in this case, virtual bystanders (an exception is the contribution of Lee and Marsella [13]). For applications that do want to offer the experience of social interaction in a virtual environment though, reports about bystanders in normal life suggest that virtual bystanders may have a relevant contribution in the experience of the social interaction. Virtual reality exposure therapy (VRET) for the treatment of social anxiety disorder is such an application, and currently receives increasing scientific and public attention [6, 9, 14, 15]. VRET is put forward as an alternative option for traditional exposure therapy in vivo because of its low cost, repeatability and convenient manipulation. In a recent controlled experiment, Anderson, Price [9] found no difference in effectiveness between VRET and in vivo exposure therapy for treating social anxiety disorder. To be effective though, these virtual environments need to be engaging enough to activate anxiety in the patients [16]. The perception of negative human evaluation during social interaction is the main component to activate patients’ social anxiety. The behavior and attitude of virtual bystanders can therefore play an important role and manipulating this may be a useful anxiety stimulus for therapists to control the intensity of patients’ anxiety level.

The current study tries to address this gap in knowledge about virtual bystanders. An experiment was conducted to examine whether bystanders’ judgments could influence a person’s beliefs, self-efficacy, and emotions during a virtual English lesson. The bystanders, i.e., virtual students, made either positive or negative comments, while other fellow virtual students or the human participant answered questions from a virtual teacher. The experiment was designed to address four hypotheses, of which the theoretical background is given below.

### 1.1 Bystander evaluation

A bystander is a person who, although present at some event, does not take part in the event, and is often regarded as an observer or spectator. Although bystanders do not get involved in the event, their behavior may influence an individual’s cognition and judgments. For example, Asch [4] investigated the effect of majority opinions on individuals and found that people often modified their judgment in accordance with the majority. The social facilitation theory also claims that the presence of other people affects individual’s performance, i.e., it enhances the individual’s performance for well-practiced tasks, but impedes it for less familiar tasks [17]. Bystanders can also have an effect on each other. A well-known phenomenon studied in this context is the so-called bystander effect, referring to the observation that people are less likely to help a victim when other people are also present. The probability that a person actually provides help is inversely related to the number of other bystanders [18]. Bystanders also play an important role in social comparison theory [19], which argues that people evaluate their abilities and opinions by comparing it with others who are similar to them. This phenomenon occurs especially in situations where the evaluation is objectively unclear [20–22]. For example, people are strongly influenced by the behavior of others when deciding whether to conserve energy in their homes [23].

Likewise, people’s perceived self-efficacy, i.e., the subjective probability that one is capable of executing a certain course of actions, has also been linked with verbal persuasion of others [24]. Evaluative feedback highlighting a person's capabilities raises efficacy [25]. Given the same level of performance, destructive criticism lowers perceived efficacy, whereas constructive criticism sustains or even boosts one’s sense of perceived efficacy [26]. Self-efficacy is a relevant concept for understanding people's behavior, since some studies have shown a strong relation between both [27, 28]. As such, self-efficacy has also been related directly or indirectly to social anxiety. For example, Alden, Teschuk [29] found that people with low self-efficacy reported that they attended more to themselves and spent more time focusing on themselves during social interaction; hence, suggesting self-efficacy to be inversely related to self-focused attention. In addition, self-focused attention is one of the key symptoms of anxiety disorder and these symptoms have been reported to correlate with each other [30]. Hope, Heimberg [31] found that socially anxious people were significantly more self-focused during social interaction than people who were not socially anxious. Other studies [32, 33] have reported a direct inverse relation between self-efficacy and social anxiety.

Only recently has the idea of virtual bystanders received attention in the context of virtual environments. For example, Kozlov and Johansen [34] were able to replicate in a virtual environment the inverse relation between the number of bystanders and the chance any person would intervene. Slater, Rovira [35] tested the response of Arsenal supporters being bystanders to a violent argument in a virtual bar. They found that when the virtual victim was an Arsenal supporter instead of a person ambivalent towards the football club, the Arsenal supporters were more likely to physically and verbally intervene in the violent argument as they shared a common social identity with the virtual victim. Park and Catrambone [36] studied social facilitation in virtual reality and found that for easy tasks people performed better in company with a virtual human than on their own, while the opposite effect was found for difficult tasks. Still, to the best of our knowledge, the effect of bystanders on individuals’ dialogue experience with a virtual human has not yet been studied empirically. Nevertheless, previous work that focused on direct interactions between a human and a virtual human has shown that a virtual audience [37] or a single virtual conversation partner [8] can effectively elicit higher or lower anxiety in a human speaker by expressing positive or negative emotions. Therefore, the current study investigates the effect of virtual bystanders expressing emotional behavior on an individual’s experience by putting forward the **first hypothesis**: *Positive compared to negative expressed attitudes by virtual bystanders towards a human speaker result in (H1a) higher self-perceived performance*, *(H1b) higher self-efficacy*, *and (H1c) less anxiety*.

### 1.2 Modeling

The social cognitive theory [38, 39] suggests that people can learn from their observations, and use learned behavior when they are in the observed situation. People are motivated by the success of others who are similar to them [38]. For example, the likelihood of learning increases when the models are of the same sex [40], skill level [41] or have similar previous behavior such as alcohol consumption [40]. People are more likely to perform the modeled behavior if it results in rewards instead of unrewarding or punishment [38]. Bandura, Ross [42] found that children who observed an aggressive model being rewarded show more imitative aggression compared to children who observed a model being punished for the same aggressive behavior.

Modeling, also referred to as vicarious experience, has also been studied in virtual reality. Fox and Bailenson [43], for example, let people observe a virtual lookalike or a dissimilar virtual person doing physical exercises. They found that either the reward of the virtual lookalike losing weight or the punishment of the virtual lookalike gaining weight was sufficient to encourage people to exercise significantly more than when observing these consequences affecting a virtual dissimilar person. However, what would happen if a person that is part of a group of bystanders, who are observing a conversation between two people, knows that he or she will be the next person having to have a conversation that will be observed by the same bystanders? As anticipation anxiety has been linked with performance anxiety [44, 45], i.e., the fear to perform in front of others, this anticipated transition from a bystander to a person being observed might lead to anticipation anxiety especially if the individual witnesses negative consequences as a bystander for persons who are similar to him or her. Bandura [24] suggests that when the vicarious experience includes positive consequences it may enhance self-efficacy. Hence, we expect that when bystanders witness positive feedback, their self-efficacy will be raised and their anxiety will be reduced. Together this leads to the **second hypothesis**: *Positive compared to negative expressed attitudes by virtual bystanders towards preceding virtual peer speakers results in (H2a) higher self-efficacy*, *and (H2b) less anxiety in a succeeding human speaker*.

### 1.3 Consistency

Modeling can affect people’s beliefs, but what happens to these beliefs if the real experience turns out to be inconsistent with the vicarious experience? That is, what happens if bystanders were positive towards peer student speakers, but later on negative towards the human speaker? In general, humans prefer consistency in behavior because of its perceptual simplicity [46]. Consistency serves the need for coherence and effective action, and it is inherent to human nature as a result of neurophysiological processes and the capacity for logical reasoning [47]. Inconsistency usually makes people psychologically uncomfortable [48]. In Festinger [48] theory of cognitive dissonance, inconsistency between two beliefs exists when holding one belief conflicts with holding the other one. Inconsistency between cognitive elements such as beliefs and items of knowledge is assumed to enhance dissonance, which motivates the individual to change one or more cognitive elements to eliminate or reduce the magnitude of the dissonance. In other words, the theory of dissonance assumes a motivation for people to maintain consistency among their beliefs, feelings and actions. When the individuals’ actions conflict with their beliefs, they are expected to try to reduce the dissonance either by changing their beliefs or by changing their behavior.

In a situation where bystanders first comment on the presentation of virtual peer speakers and later on a human speaker, inconsistency in these comments may force the human to change his or her belief much more extremely, than when the bystanders express exclusively positive or negative comments in both occasions. This leads to the **third hypothesis** (*H3*): *Inconsistency in the bystanders’ expressed attitude towards virtual peer speakers and the human speaker leads to a larger change in belief than consistency in the bystanders’ expressed attitudes*.

### 1.4 Praise and destructive criticism

Up till now, the focus has only been on how bystanders affect beliefs that people have about themselves. However, bystanders may also affect beliefs that people have about the bystanders. For example, accumulated findings in the form of a meta-analysis [49] support the claim that flattery has a positive influence on people’s judgment of the flatterer. The self-enhancement motive, i.e., people are motivated to evaluate themselves favorably and they respond positively by increased liking for people who flatter them, is suggested as a crucial factor underlying the positive effect of flattery [49, 50].

Reeves and Nass [51] also studied the effect of text-based flattery by computers and found that individuals who were flattered by the computer performed better and liked the computer more than individuals who received no feedback or criticism from the computer. Johnson, Gardner [52] found that their participants reacted to flattery from a computer in a manner congruent with peoples’ reactions to flattery from other humans, but only for participants with a high level of computer experience. Consistently, Lee [53] found that flattery led to more positive overall impressions and performance evaluations of the computer. It seems therefore that bystanders’ comments could also affect the beliefs people have about them. This therefore leads to the **fourth** and final **hypothesis** (*H4)*: *Beliefs about the bystanders correlate positively to the attitude bystanders express towards the human speaker*.

## Method

An experiment with a two-by-two within-subjects design existing of four conditions (as shown in Table 1) was setup to test the four hypotheses. It included two within-subjects factors: (1) the virtual bystanders’ attitude towards the virtual student speakers who answered questions before the human speaker’s (i.e., the participant) turn to answer questions, and (2) the bystanders’ attitude towards the human speaker. The bystanders’ attitude could be either positive or negative, i.e., whispering either positive or negative remarks towards other bystanders and showing an angry or happy facial expression. Participants were exposed to all four conditions. To control for potential learning, order or fatiguing effects, the order of the four conditions was counterbalanced.

**Table 1:** Four experimental conditions with a different attitude of the virtual bystanders towards the virtual peer speakers and the participants.

Condition	Bystanders’ attitude towards virtual peer speakers (phase 1)	Bystanders’ attitude towards the human (phase 2)
*PP*	Positive	Positive
*NP*	Negative	Positive
*PN*	Positive	Negative
*NN*	Negative	Negative

### 2.1 Ethics statement

The experiment was approved by the Human Research Ethics Committee of the Delft University of Technology. Written informed consent was obtained from all participants prior to the experiment. Furthermore, for publication policy, the individual in this manuscript has also given written informed consent (as outlined in PLOS consent form) to publish case details. All participants received a small gift for their contribution.

### 2.2 Participants

Participants were recruited through advertisements spread over the university campus. Twenty-six students (9 females and 17 males) from Delft University of Technology participated in the experiment. Their age ranged from 20 to 30 years with the mean being 26.8 (*SD* = 2.5) years. All participants were non-native English speakers (i.e., 10 Chinese, 5 Dutch, 3 Romanian, 2 Greek, 1 Danish, 1 Indonesian, 1 Mexican, 1 Portuguese, 1 Spanish and 1 Syrian). To get admission to the university, these students all passed one of the following criteria: an overall Band score of at least 90 on the “Test of English as a Foreign Language (internet-based)”, an overall Band score of at least 6.5 on the IELTS (academic version), or a proof of having passed the University of Cambridge “Certificate of Proficiency in English” or the University of Cambridge “Certificate in Advanced English”. Apart from this criterion on English proficiency, no further exclusion criteria were used for the recruitment of the participants. They also were all naive with respect to the hypotheses until they finished the experiment.

### 2.3 Measurements

The construct perceived performance, put forward in the hypotheses, was operationalized by considering the following indicators: (1) participants’ rating of their own, virtual peers’ and the teacher’s performance, and (2) satisfaction with their own, virtual peers’ and the teacher’s performance. Anxiety was measured subjectively through the subjective units of discomfort (SUD) scale [54], physiologically, through skin conductance and heart rate, and behaviorally through speech length. In addition, the Personal Report Confidence as a Speaker (PRCS) questionnaire [55] and the Igroup presence questionnaire (IPQ) [56] were used to measure the participants’ general social anxiety and presence experienced in the virtual environment.

#### 2.3.1 Personal Report of Confidence as a Speaker

The Personal Report of Confidence as a Speaker (PRCS) questionnaire [55] was used as a screening test for everyday experienced fear of speaking. It is a self-report questionnaire that assesses the behavioral and cognitive response to public speaking. It recorded whether participants agreed or disagreed (i.e., a binomial response) on 30 statements, for example “I dislike to using my body and voice expressively.” The PRCS index was scored by counting the number of answers indicating anxiety. The PRCS index ranged from 0 to 30. Daly [57] reported that PRCS had a good internal consistency (Cronbach’s *α* = .91) and it was strongly positively correlated with other social phobia measures. Furthermore, Phillips, Jones [58] showed no difference across age, gender and race in the PRCS index.

#### 2.3.2 Presence questionnaires

Participants’ sense of presence was also measured as recently a meta-analysis showed that anxiety experienced in a virtual environment is associated with presence [59]. Participants were asked to complete the Igroup Presence Questionnaire (IPQ) [56] at the end of the experiment to measure their experienced presence during the exposure in the virtual environment. IPQ consisted of 14 items rated on a seven-point Likert Scale. The scores on the 14 IPQ items were mapped onto three subscales, namely Involvement (i.e., the awareness devoted to the virtual environment), Spatial Presence (i.e., the relation between the virtual environment and the physical real world), and Realism (i.e., the sense of reality attributed to the virtual environment). The questionnaire also contained one item that assessed the general feeling of being in the virtual environment. The total score of IPQ was used in the data analysis to test whether the level of presence was sufficient to evoke an emotional response in the participants. The total score of IPQ ranged from 0 to 84. The IPQ questionnaire had a good internal consistency (Cronbach’s *α* = .87), and validity, i.e., a high correlation with other presence measures was repeatedly found [10].

Recently, Slater [60] argued that presence at least has two independent components: place illusion and plausibility. Similar to physical presence, place illusion refers to the feeling of being in the virtual environment. Plausibility is the illusion that what is happening in the virtual world is really happening in spite of the knowledge that it is mediated technology. A high level of plausibility would elicit responses in the virtual environment similar to the ones in the real world. For VRET for social anxiety disorder, plausibility may be more relevant than place illusion. Therefore, for this experiment participants were asked to complete a newly created presence response scale (PRS), focusing on plausibility, using the following three items: (1) How often did you find yourself automatically behaving within the virtual English class as if it were a real English course? (2) To which extent was your overall behavior (what you said, emotional response and thoughts) like being in a real English course? (3) How much did you feel like being in a real English course? The PRS questionnaire had good reliability with Cronbach’s *α* ranging from. 88 to. 92 across the four conditions in this study.

#### 2.3.3 Belief and experience questionnaire

The belief and experience questionnaire (BEQ), of which all items are shown in S1 Text, was specifically made for this experiment and used to measure participants’ beliefs about their own performance and that of the virtual peers and the teacher. The questionnaire also included questions with regards to satisfaction towards the performance of themselves, the virtual peers and the teacher, as well as questions related to the supportiveness of the virtual peers and the teacher, and self-efficacy. The formulation of self-efficacy question was based on the one used in a study by Kashdan and Roberts [32]. All items were measured on scales ranging from 0 to 10.

The participants’ experience of the lesson was measured on six semantic differential scales including unpleasant—pleasant, not relaxed—relaxed, aggressive—non-aggressive, uncomfortable—comfortable, impolite—polite and exhausting—energizing. All scales ranged from 0 to 10. The average score across the six scales was taken as an index for a participant’s experience. The questionnaire measuring participants’ experience in the virtual lesson had good reliability with Cronbach’s *α* ranging from. 71 to. 88 across the four conditions.

#### 2.3.4 Subjective units of discomfort

The 11-point scale of subjective unit of discomfort (SUD) was used to measure the perceived level of anxiety of the participants. A score of 0 represented no fear and a score of 10 represented the highest level of fear an individual has ever felt in his or her life [54].

#### 2.3.5 Physiological measurements

Physiological measurements, including heart rate and skin conductance, were included to measure elicited arousal during the virtual English lesson. The physiological measurements were done with a Mobi8 system from TMSi. To measure skin conductance, two finger electrodes were used. Heart rate was recorded using an Xpod Oximeter, and the participants were requested to insert a finger into an adult articulated finger clip sensor. An elevation in heart rate or skin conductance was regarded as an indicator for increased arousal.

#### 2.3.6 Speech length

Speaking time was suggested as a reliable behavioral measure to assess performance anxiety [61]. In an impromptu speech task, patients were asked to give a speech, and the length of the speech was taken as a reversed indicator of avoidance behavior. Anderson, Price [9] also used the length of a participant’s speech as a behavioral avoidance measure. Therefore, in this experiment the total time a participant talked during the discussion was recorded as an indicator of engagement, or reversed, of avoidance caused by anxiety.

### 2.4 Apparatus

**Fig 1:**
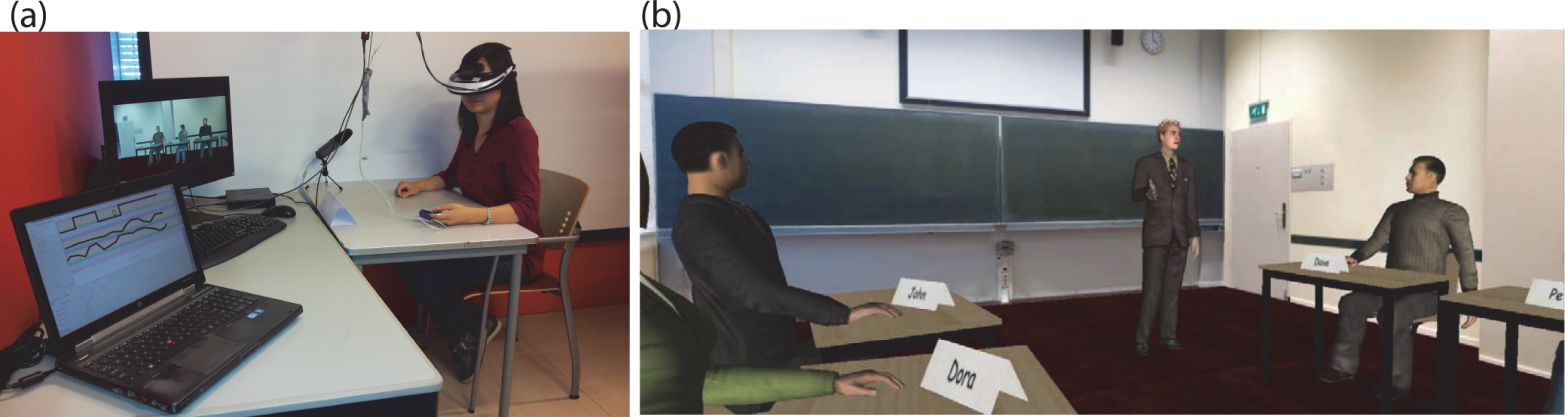
The experimental setup with (a) a participant doing the experiment, and (b) the participant’s view of the virtual environment.

As shown in Fig 1, the virtual environment was displayed non-stereoscopically on a Sony HMZ-T1 Head-Mounted Display (HMD, 1280 × 720 pixels with 51.6° diagonal field of view) coupled to a three-degrees of freedom head tracker with a 500Hz update rate. Participants could freely look around to explore the virtual classroom, shown by means of a screenshot in Fig 1B. Sound was played through embedded headphones. Besides the HMD, the participants wore a finger clip and two finger electrodes on their non-dominant hand for the Mobi8 system to record physiological data, including heart rate and skin conductance.

**Fig 2:**
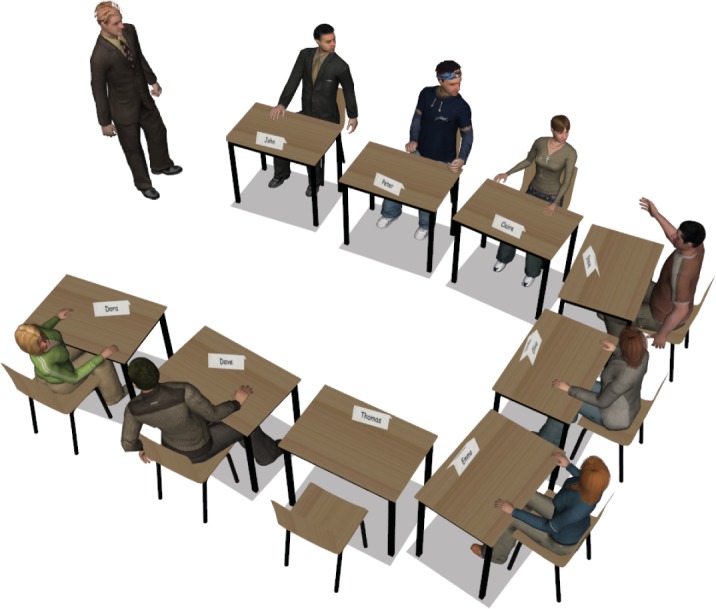
The layout of the virtual classroom where the participant was seated on the empty chair.

The virtual environment was created using WorldViz’s Vizard 3.0, and recreated an English lesson where a teacher asked students in turn general questions to practice their English conversation skill. Besides the participant, there were eight virtual fellow students sitting in the classroom: four males and four females. The classroom layout is shown in Fig 2. The participant was always sitting on the third desk at the left side, while the position of the other virtual students was randomly assigned in each condition. The clothes and hair of the virtual students were always different in the four conditions to create the impression that each condition involved a different set of students.

In front of each virtual student was a name card, so the participant knew who was addressed by the teacher. The name of the participant was always ‘Thomas’ or ‘Mary’, depending on the gender of the participant. There was also a name card on the desk of the participant in real life to remind him or her of the temporary name in the virtual environment. The teacher was a well-dressed male aged around 40 to 50 years old. As the experiment focused on the virtual students, i.e., the bystanders, and not on the virtual teacher, i.e., the primary communication partner of the participants, the appearance and the neutral attitude of the teacher was the same across the conditions. The voice actor of the teacher was a native English speaker, while the voice actors/actresses of the students were all non-native English speakers. A total of 28 open questions were recorded, seven for each condition. The teacher first posted four questions to different virtual students, randomly; when the last virtual student finished answering the fourth question, the teacher asked the participant to answer that same question again, saying “Thomas/Mary, how about you?” and after that the last remaining three questions were all asked to the participant. The questions included examples as “What is the one thing that disgusts you and why?”, “If you could go anywhere right now, where would that be and why?” and “What is the worst thing about being a grown-up and why?”. So, these questions were formulated such that they had no clear objective evaluation criteria for the answers. All the eight voice actors/actresses of the virtual students were recorded while answering these questions spontaneously. So the teacher could ask anyone of the virtual students to answer his questions.

**Fig 3:**
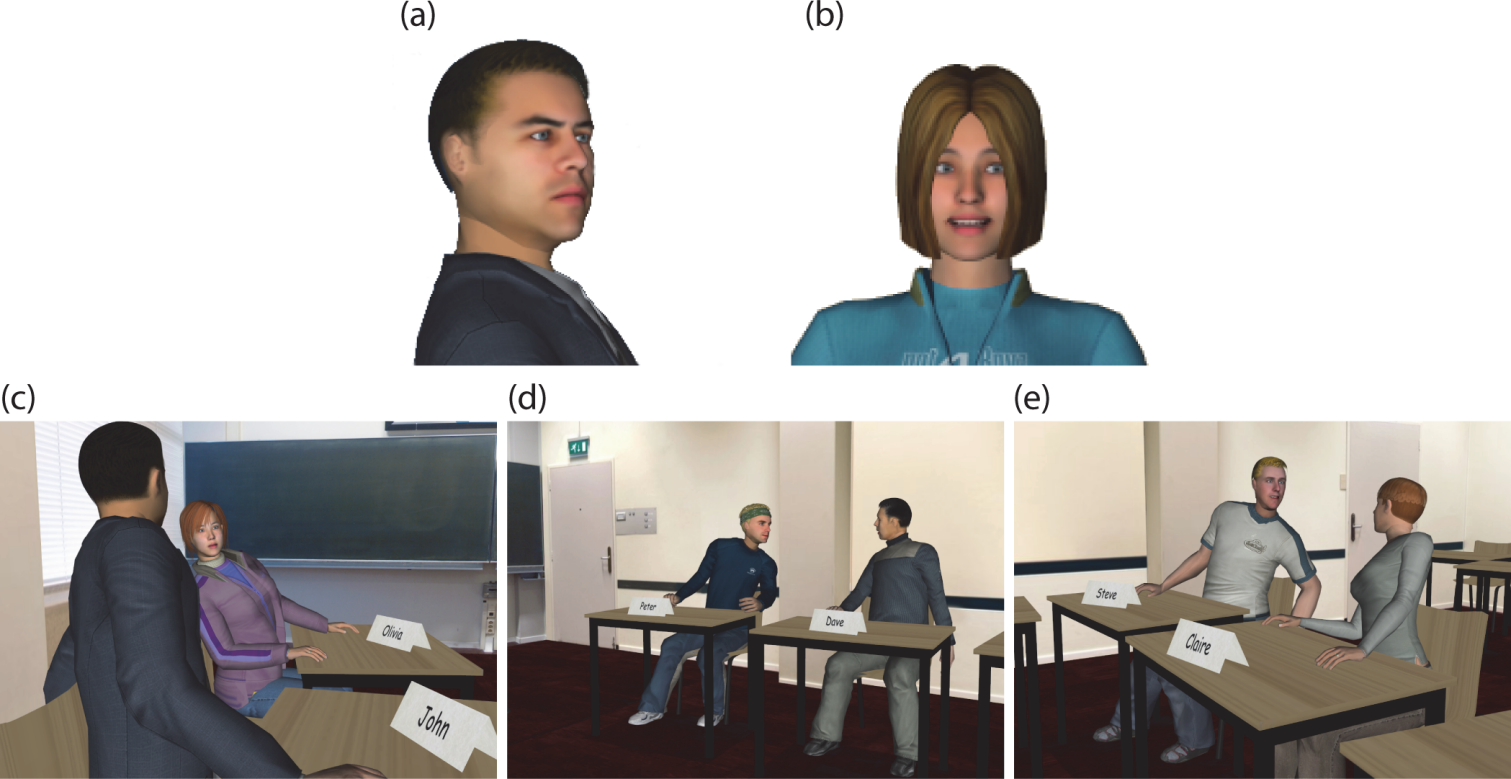
Facial expressions used in the experiment and virtual students (bystanders) whispering to each other (screenshots). (a) a virtual student showing an angry facial expression, (b) a virtual student showing a happy facial expression, (c) students whispering at the participant’s left side, (d) students whispering in front of the participant, and (e) students whispering at the participant’s right side.

Only the virtual bystander students were able to show positive or negative behavior in the different experimental conditions. This behavior mainly consisted of facial expressions and whispering to each other, as illustrated in Fig 3. Two facial expressions were used in this experiment: angry (see Fig 3A) and happy (see Fig 3B) to express negative or positive behavior respectively. The facial expression was achieved by a repeatable facial expression animation method, explained and evaluated in a previous study by Broekens, Qu [62]. Different attitudes of the students were also expressed in their whispers. In the positive condition, virtual students whispered positively to each other; for example, one student would say “Hey, this is a good answer!” and another bystander student would reply “Yes, a good one!”, or the first student would say “I like it!”, and another bystander would respond “I also like it!”. In the negative condition, their whispers had a negative connotation; for example, one bystander student could say “I don’t like the answer!” and another student would replied “Me neither!”, or a student would say “Boring!” and another student would reply “Yes, so boring!”. So, all whispers focused on the content of the answers, and did not focus on the English formulation of the answer. A total of 36 pairs of positive or negative whisper dialogs were recorded for each of the eight virtual students, so participants could see whispering students on the left (Fig 3C) or right (Fig 3E) of them or in front of them (Fig 3D). The whispers were designed to occur every 6–10 seconds after the virtual students or the participant started answering questions from the teacher. The questions towards the participants were triggered using speech detection. Three consecutive seconds of silence after the participant’s answer triggered the teacher to give a neutral response such as ‘ok’ or ‘all right’, after which he would use a transition phrase such as ‘next’ or ‘the next question’ to introduce the next question. The speech detection was also designed to handle the situation that a participant would not say anything after the teacher posed a question. After 3 seconds of silence, the teacher would repeat the question and ask the participant to answer it again. In addition, to prevent a participant to give a very short answer to some of the questions, such as ‘I don’t know’, the teacher would ask ‘why’ if the participant’s answer was shorter than 5 seconds. An example of the interaction in the virtual classroom can be seen in S1 Video.

### 2.5 Procedure

Prior to the experiment, participants were provided with an information sheet, and the procedure was explained to them. They were then asked to sign an informed consent form, and to fill in a general information questionnaire, including SSQ and PRCS (see section Measurements for more details). There were two phases in each condition: in the first phase, the virtual English teacher asked four virtual peer students a question, and in the second phase, the teacher asked the participant four questions. The virtual bystanders’ attitudes towards the virtual peer speakers and towards the participant were manipulated as either positive or negative according to Table 1. At the start of each condition, a pre-SUD score was obtained, whereas the BEQ, a post-SUD score and the PRS were administered at the end of each condition. After the participants experienced all the four conditions, the IPQ and SSQ were administered. Heart rate, skin conductance, and the length of a participant’s answers were recorded during the experiment. The experimenter left the experimental room when a session started. Afterwards there was a debriefing session, in which the experimenter and the participant discussed the experiences and the experimenter explained to the participant the full details of the experiment. The whole experiment took about 50 minutes.

### 2.6 Data preparation and statistical analyses

For each condition participants completed the BEQ, PRS, pre-SUD and post-SUD questionnaires. This resulted in a set of 18 dependent variables expressing participants’ beliefs about themselves (P1-P7), their self-efficacy (P8), their anxiety (P9-P10), and their judgments on the virtual other students (S1-S4) and the teacher (T1-T4) (see also S1 Text). The labels given here to the various dependent variables are consistently used in the various tables and in the remainder of the text of this paper. To reduce variation caused by individual differences, the data for the dependent variables were first standardized into *z*-scores for each participant across all items of a questionnaire and the four conditions. Likewise, the total speech length of participants’ answers and the length of the answer to the first question were first standardized into *z*-scores for each participant across the four conditions. In contrast to the other data we collected, the physiological data also provided information when the participants were observing the virtual peer students answering questions. Therefore, we split our physiological data into two phases: the peer answering phase and the participant answering phase. The data on skin conductance were standardized into *z*-scores for each participant across eight moments, i.e., the two phases in each of the four conditions. As heart rate data was normally distributed, the analysis was conducted on the original data. For one participant the physiological data were lost due to technical problems.

Next, the effect of pre-experimental differences was evaluated by calculating correlations between, on the one hand, the pre-SUD scores and the PRCS scores, and, on the other hand, the dependent variables BEQ, PRS, post-SUD and its difference with the pre-SUD, speech length, heart rate, and skin conductance for all the four conditions. No significant correlations were found, and therefore, these results did not warrant analyses with pre-SUD or PRCS as covariates [63, 64].

To avoid inflated Type-I error, a three-steps strategy with omnibus tests was followed for the statistical analyses. First, four doubly multivariate repeated-measures analysis of variance [65] were used to test each of four hypotheses on an overall level for the multiple dependent variables. The analyses for the first, second and fourth hypothesis used a full factorial design, using two within-subject variables: *peers*, i.e., virtual bystanders’ attitude towards the virtual student speakers; and *participants*, i.e., virtual bystanders’ attitude towards the human speaker. The third hypothesis, about (in)consistency, only used one within-subject factor, i.e., *consistency*. To do so, the results of the four conditions (see Table 1) were combined into a consistent condition, i.e., (*PP*-*NN*)^2^, and an inconsistent condition, i.e., (*NP*-*PN*)^2^. In the second analysis step, univariate repeated-measures analyses of variance on individual measures were conducted to test effects that were found to be significant in the overall analysis. The third step consisted of paired *t*-tests to examine significant two-way interaction effects which were found in the previous omnibus tests. Note that the results in subsequent steps were ignored if the previous step didn't indicate significance.

Interaction style has been reported to differ between same-gender (e.g., male students interacting with a male teacher) and mixed-gender (e.g., female students interacting with a male teacher) [66]. Therefore, we also repeated our analyses including only the 17 male participants. In addition, we repeated the analyses also including participants’ gender as a between-subject variable, since previous literature [67] reported that females increased their participation by giving comments in a virtual class compared to a traditional class.

## Results

All the experimental data are available in S1 Table. Our participants all met the English requirement of the university, and most of them rated their English level as average, except for two participants who rated their English as good. The mean and standard deviation of the PRCS scores over all 26 participants were *M* = 8.62, *SD* = 5.0, indicating that the participants included in the experiment were generally socially confident. Also no significant difference was found between male (*M* = 8.00, *SD* = 4.53) and female (*M* = 9.78, *SD* = 5.91) participants’ PRCS (*t*(24) = .86, *p* = .40, *d* = 0.35). In addition, no significant difference was found between male (*M* = 2.08, *SD* = 2.09) and female (*M* = 3.22, *SD* = 2.31) participants’ SUD score tested at the beginning of the experiment, i.e., pre-SUD score of the first condition (*t*(24) = 1.28, *p* = .21, *d* = 0.52). The IPQ data from the 26 participants suggested that a reasonable level of presence was obtained in the experiment as no significant difference was found between the IPQ total score of the online dataset [68] for non-stereoscopic HMD (*M* = 45.73, *SD* = 7.98) and the IPQ total score in the current experiment (*M* = 46.35, *SD* = 9.86) using an independent-samples *t*-test (*t*(35) = 0.18, *p* = .86, *d* = 0.06).

Table 2 shows the results of the overall analyses for the effect of two factors, *peers* and *participant*, on the related measures per hypothesis. The results showed support for the virtual bystander effect, i.e., the first hypothesis. The bystanders’ attitudes towards the participants affected the participants’ performance beliefs, self-efficacy or their anxiety significantly. The analysis also showed a significant two-way interaction between bystanders’ attitudes towards peer speakers and participants. The overall analysis also found support for the modeling mechanism of the preceding virtual peer speaker, i.e., the second hypothesis. For the self-efficacy or anxiety measures, a significant two-way interaction, but no significant main effect for bystanders’ attitude towards peer speakers was found. Neither did we find support for the (in)consistency in peer speakers' attitudes on changes in participants’ beliefs, i.e., the third hypothesis. The overall analysis did however find support for a reciprocal flattering effect on beliefs about the bystanders (or the teacher), i.e., fourth hypothesis. The results indicated a significant main effect for bystanders’ attitude towards participants.

**Table 2:** Results of the repeated-measures MANOVAs on the overall effect of virtual bystanders’ attitudes towards the virtual peer speakers and the participants for the four hypotheses.

		Degree of freedom				
Hypothesis and dependent variables	Independent variables	Hyp	Err.	*F*	*p*	*η^2^*
**H1**: Self-perceived performance (P1-P7), Self-efficacy (P8), Anxiety (SUD post (P9) and SUD post-pre (P10), Speech length, Heart rate, and skin conductance	Participant	13	12	9.13	**<.001**	**.91**
	Peers	13	12	1.29	.334	.58
	Participants × Peers	13	12	5.02	**.004**	**.85**
**H2**: Self-efficacy (P8), Anxiety (SUD post (P9) and SUD post-pre (P10), Speech length, Heart rate, and skin conductance)	Participant	6	19	4.89	**.004**	**.61**
	Peers	6	19	1.32	.296	.29
	Participants × Peers	6	19	6.51	**.001**	**.67**
**H3**: Self-perceived performance (P1-P7), Self-efficacy (P8), Attitudes towards the virtual bystander (S1-S4)and the teacher (T1-T4)	(In)consistency	16	10	1.91	.150	.75
**H4**: Attitudes towards the virtual bystander (S1-S4) and the teacher (T1-T4)	Participant	8	18	33.12	**<.001**	**.94**
	Peers	8	18	0.97	.492	.30
	Participants × Peers	8	18	1.60	.194	.42

The results in Table A in S2 Table show that some findings no longer reached a significant level when the analyses were conducted on the male only sample. This might be caused by the reduced sample size, and the related lower statistical power. The results in Table A in S3 Table show no additional significant interaction between gender and bystanders’ attitudes towards either the participants or the peer speakers.

### 3.1 Self-reported belief, experience, and anxiety

**Fig 4:**
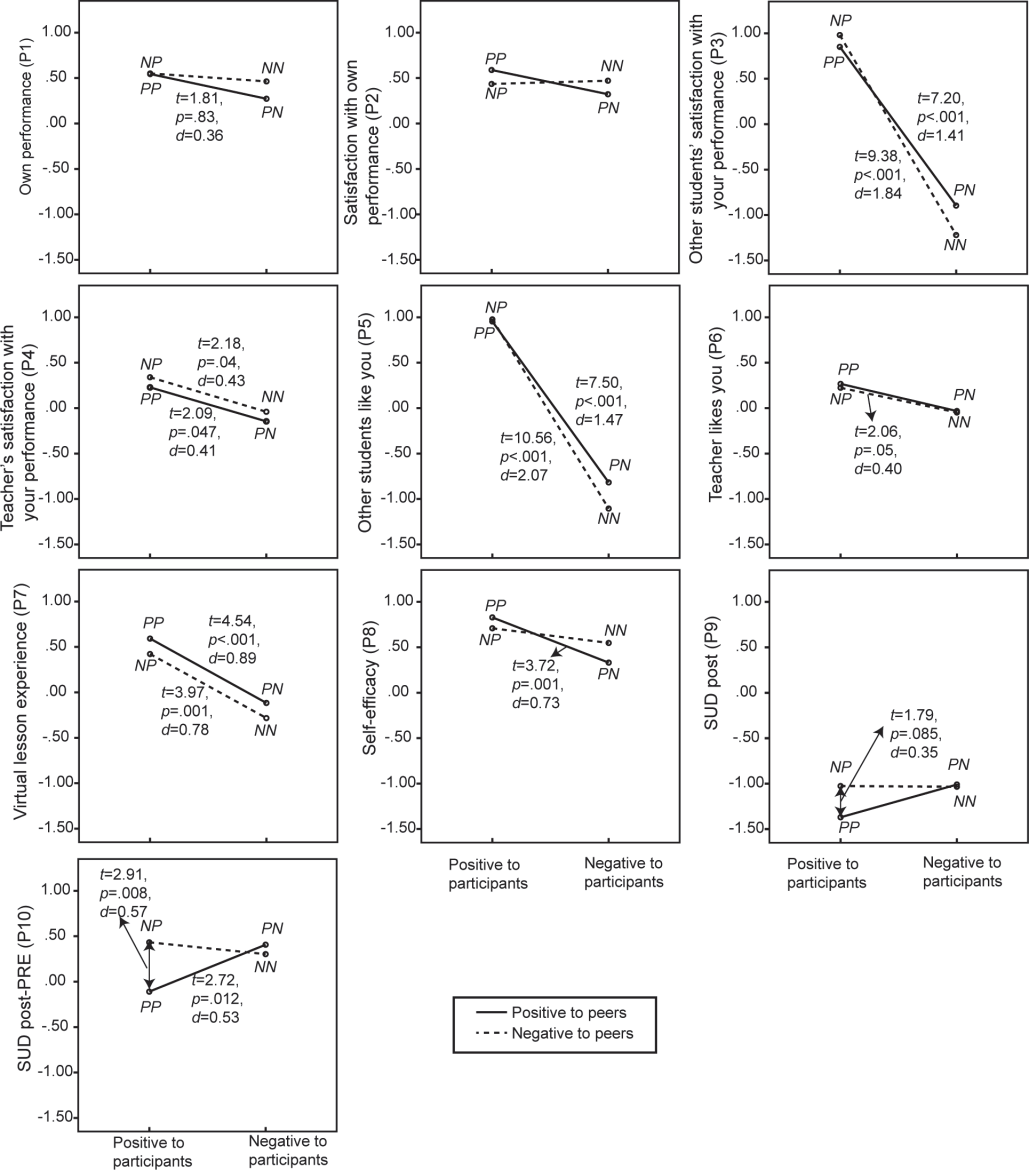
Results of the participants’ self-related belief and experience questionnaire, and self-reported anxiety, including results of paired *t*-tests (*df* = 25).

Mean and standard deviation of the 18 dependent variables for the four conditions are shown in Table 3. Table 4 shows the results of 18 univariate repeated-measure ANOVAs on BEQ and self-reported anxiety. The first group of BEQ items (P1—P8) was about beliefs related to participants. The bystanders’ positive attitude towards the participant compared to the conditions where the bystanders exhibited a negative attitude towards the participant, resulted in participants believing that peers and teacher were significantly more satisfied with their performance (P3 and P4) and liked them significantly more (P5 and P6), and resulted for the participants in a significantly more positive lesson experience (P7) and significantly more self-efficacy (P8). Although no significant effect for the bystanders’ attitude on self-perceived performance (P1) and on the participants’ satisfaction with their own performance (P2) was found, the *p*-value of 0.067 for the self-perceived performance (P1) approached the significant threshold of α = 0.05. The two-way interaction found by the overall analysis for first and second hypothesis was found back in the univariate analysis of the reported self-efficacy (P8), as illustrated in Fig 4. When initially the bystanders showed a positive attitude towards the virtual peer speakers, the participants’ self-efficacy was significantly (*t*(25) = 3.72, *p* = .001, *d* = 0.73) lower if the bystanders’ attitude became negative instead of remaining positive when the participant was talking. However, when the bystanders initially showed a negative attitude towards the virtual peer speakers, no significant difference (*t*(25) = 1.71, *p* = .099, *d* = 0.34) was found in participants’ self-efficacy between conditions where the bystanders remained exhibiting a negative attitude or changed into exhibiting a positive attitude when the participant was talking.

**Table 3:** Mean and standard deviation of items of the BEQ and self-reported anxiety for the four experimental conditions.

Measurements		*PP*	*NP*	*PN*	*NN*
**The participants**					
	P1 Own performance	0.54 (0.66)	0.55 (0.63)	0.27 (0.78)	0.46 (0.64)
	P2 Satisfaction with own performance	0.59 (0.63)	0.44 (0.72)	0.32 (0.83)	0.47 (0.76)
	P3 Other students’ satisfaction with your performance	0.85 (0.71)	0.98(0.69)	-0.90 (0.88)	-1.22 (0.73)
	P4 Teacher’s satisfaction with your performance	0.23 (0.58)	0.34 (0.71)	-0.15 (0.73)	-0.04 (0.56)
	P5 Other students like you	0.95 (0.65)	0.98 (0.77)	-0.82 (0.83)	-1.11 (0.67)
	P6 Teacher likes you	0.27 (0.57)	0.22 (0.74)	-0.03 (0.65)	-0.05 (0.63)
	P7 Virtual lesson experience	0.59 (0.49)	0.42 (0.78)	-0.12 (0.60)	-0.28 (0.71)
	P8 Self-efficacy	0.83 (0.47)	0.71 (0.73)	0.33 (0.81)	0.55 (0.62)
	P9 SUD-post	-1.37 (0.94)	-1.03 (1.31)	-1.01 (1.15)	-1.03 (1.01)
	P10 SUD post—SUD pre	-0.11 (0.80)	0.43 (0.95)	0.41 (0.59)	0.30 (0.74)
**Other students**					
	S1 Other students’ performance	0.46 (0.83)	0.42 (0.68)	-0.25 (0.83)	-0.17 (0.96)
	S2 Participants’ satisfaction with other students’	0.44 (0.57)	0.38 (0.51)	-0.28 (0.78)	-0.40 (0.88)
	performance				
	S3 Participants liking the other students	0.21 (0.70)	0.27 (0.62)	-0.51 (0.86)	-0.90 (0.85)
	S4 How supportive were the other students towards you	1.00 (0.74)	1.27 (0.60)	-1.29 (0.69)	-1.53 (0.59)
**The teacher**					
	T1 The teacher’s performance	0.080 (0.62)	0.16 (0.64)	-0.10 (0.70)	-0.14 (0.67)
	T2 Participants’ satisfaction with teacher’s performance	0.11 (0.65)	0.27 (0.46)	-0.09 (0.68)	- 0.28 (0.61)
	T3 Participants liking of the teacher	0.14 (0.75)	0.05 (0.76)	-0.32 (0.82)	-0.33 (0.68)
	T4 How supportive was the teacher towards you	-0.006 (0.82)	-0.09 (0.85)	-0.15 (0.84)	-0.50 (0.78)

**Table 4:** Results of the repeated-measures univariate ANOVAs on items of the BEQ and self-reported anxiety.

Measurements	Attitude towards								
	Participant			Peer speakers			Participant×Peer speakers		
	*F*(1,25)	*p*	*η^2^*	*F*(1,25)	*p*	*η^2^*	*F*(1,25)	*p*	*η^2^*
**The participants**									
P1 Own performance	3.66	.067	.13	1.07	.31	.04	0.73	.40	.03
P2 Satisfaction with own performance	0.81	. 38	.03	0.001	.98	<.001	2.70	.11	.10
P3 Other students’ satisfaction with your performance	95.25	**<.001**	**.79**	0.97	.33	.04	3.21	.085	.11
P4 Teacher’s satisfaction with your performance	12.03	**.002**	**.33**	0.88	.36	.03	<. 001	.98	<.001
P5 Other students like you	97.53	**.001**	**.80**	1.08	.31	.04	2.60	.12	.09
P6 Teacher likes you	6.85	**.015**	**.22**	0.068	.80	.003	0.014	.91	.001
P7 Virtual lesson experience	23.45	**<.001**	**.48**	2.86	.10	.10	<.001	.98	<.001
P8 Self-efficacy	14.31	**.001**	**.36**	0.20	.66	.008	4.87	**.037**	**.16**
P9 SUD post	1.29	.27	.05	2.38	.14	.09	1.53	.23	.06
P10 SUD post- SUD pre	1.67	.21	.06	3.79	.063	.13	6.40	**.018**	**.20**
**Other students**									
S1 Other students’ performance	13.18	**.001**	**.35**	0.034	.86	.001	0.25	.62	.01
S2 Participants’ satisfaction with other students’ performance	22.24	**<.001**	**.47**	0.66	.42	.026	0.125	.73	.005
S3 Participants liking the other students	29.90	**<.001**	**.55**	2.32	.14	.09	3.97	.057	.14
S4 How supportive were the other students towards you	269.56	**<.001**	**.92**	0.028	.87	.001	6.59	.017	.21
**The teacher**									
T1 The teacher’s performance	4.82	**.038**	**.16**	0.11	.75	.004	0.38	. 55	.015
T2 Participants’ satisfaction with teacher’s performance	7.76	**.01**	**.24**	0.012	.91	<.001	4.11	.054	.14
T3 Participants liking the teacher	11.86	**.002**	**.32**	0.24	.63	.01	0.18	.67	.007
T4 How supportive was the teacher towards you	4.32	**.048**	**.15**	3.21	.085	.11	1.45	. 24	.055

Table 4 also shows the results of two repeated-measures univariate ANOVAs on the self-reported anxiety at the end of a session (P9, SUD post), and the change in self-reported anxiety (P10, SUD post—SUD pre). The analyses revealed a significant interaction effect in the change in self-reported anxiety (P10), as also illustrated in Fig 4. Participants reported significantly less change in anxiety in the condition where bystanders’ attitude was positive towards both the virtual peers and the participant compared to all three other conditions, i.e., (1) negative attitude towards peers and positive towards participant (*t*(25) = 2.91, *p* = .008, *d* = 0.57), (2) positive towards peers and negative towards participant (*t*(25) = 2.72, *p* = .012, *d* = 0.53), or negative towards both peers and participant (*t*(25) = 3.01, *p* = .006, *d* = 0.59).

**Fig 5:**
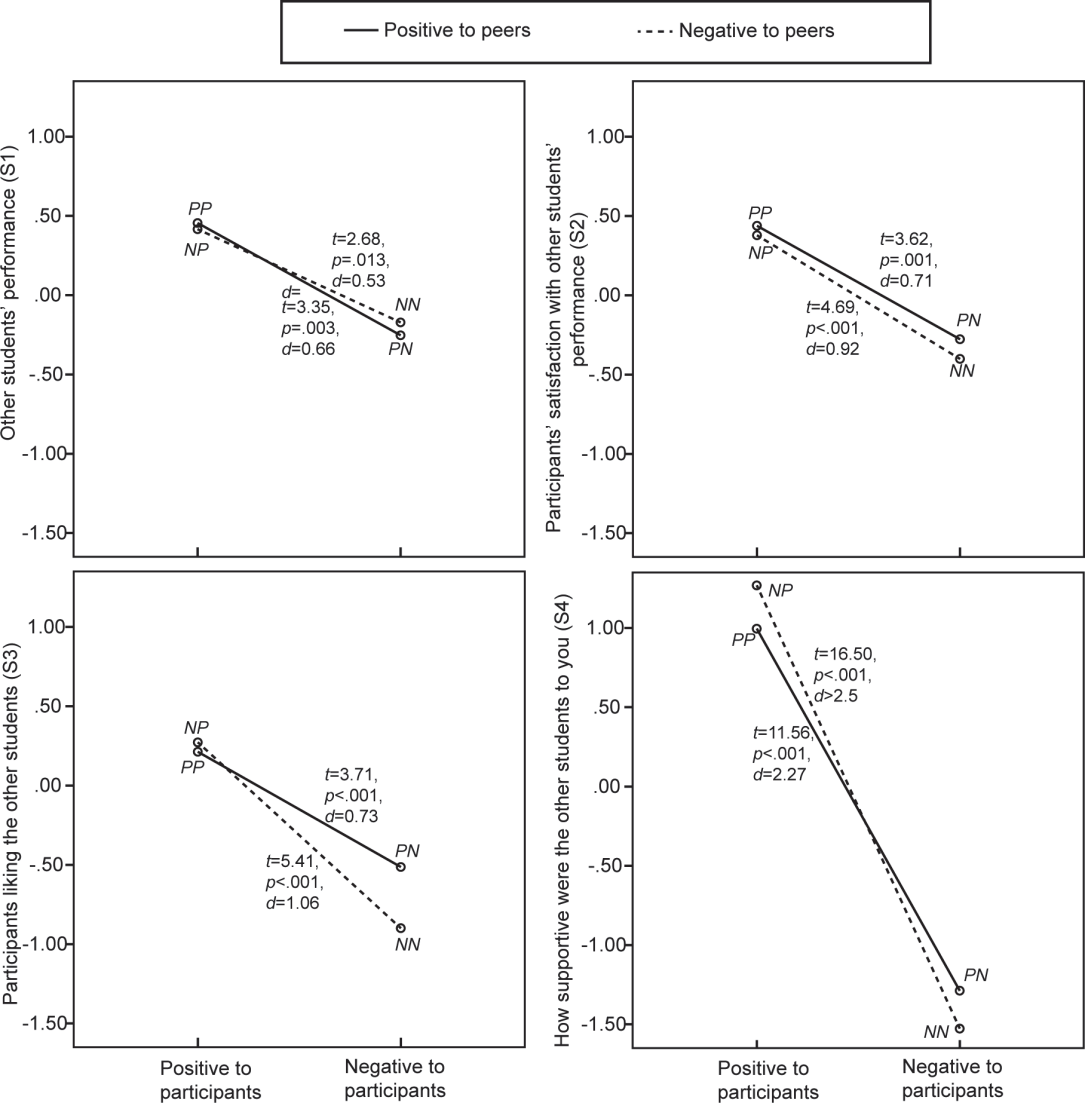
Participants’ ratings of their beliefs regarding the virtual peers, including results of paired-samples *t*-tests (*df* = 25).

**Fig 6:**
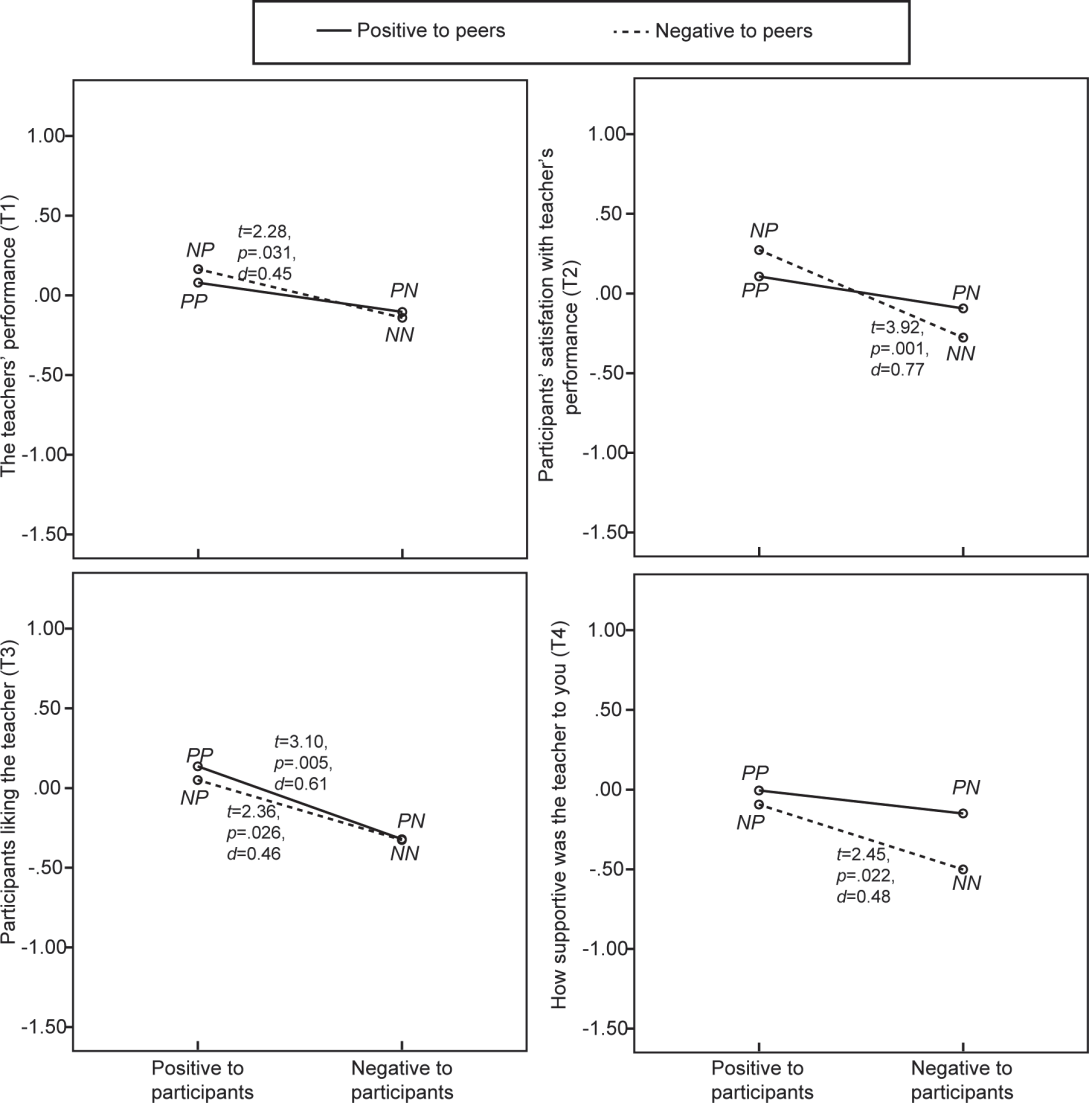
Participants’ ratings of their beliefs regarding the teacher, including results of paired-samples *t*-tests (*df* = 25).

Besides focusing on their own, the BEQ also included questions about beliefs held towards bystanders (S1-S4) and teachers (T1-T4). Table 4 shows results of univariate analysis and they are further illustrated in Figs. 5 and 6. The results showed that bystanders’ positive instead of negative attitude towards the participants resulted in significantly higher ratings for virtual peers’ (S1) and teacher’s (T1) performance, participant’s satisfaction with these performances (S2 and T2), how much the participants liked them (S3 and T3) and their supportiveness towards the participant (S4 and T4). No significant main effect for bystanders’ attitude towards the virtual peer speakers was found on any of these items.

No additional significant effects were found when the analysis was repeated including only the male participants (Table B in S2 Table) or when including participants’ gender as a between-subject factor (Table B in S3 Table).

### 3.2 Presence response scale

**Fig 7:**
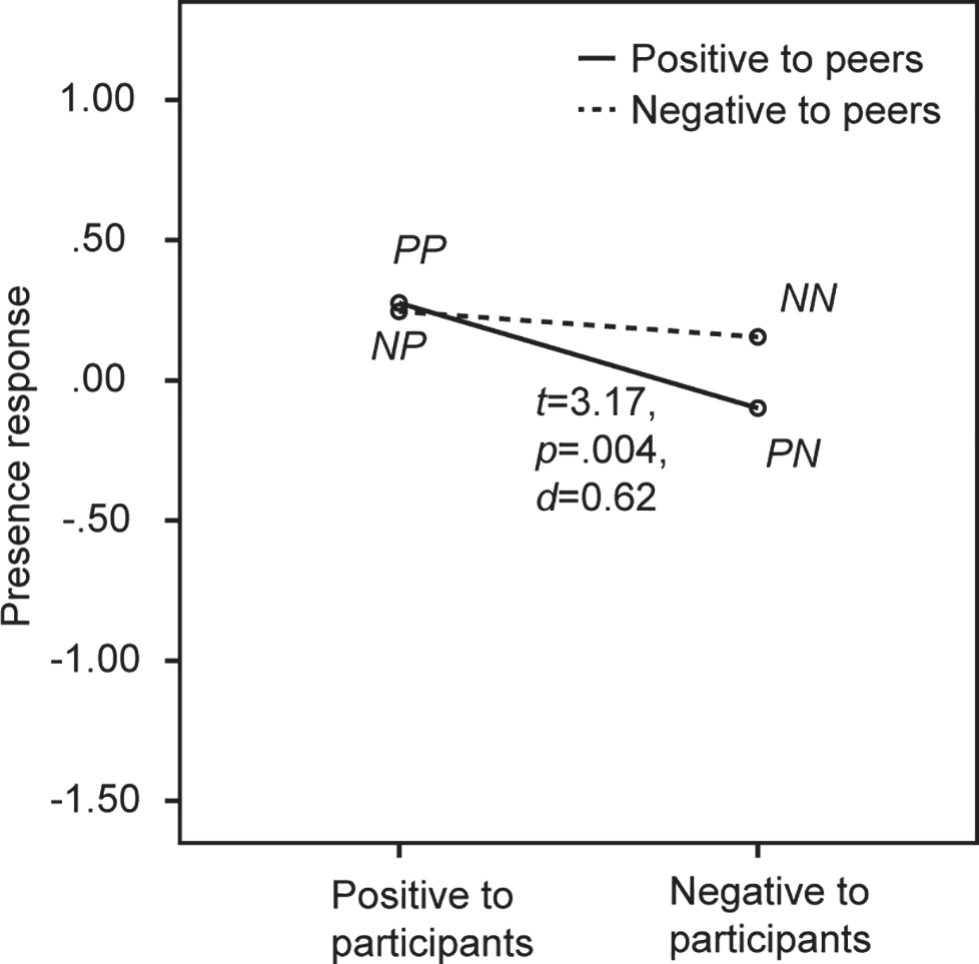
Participants’ ratings of the presence response scale, including the result of a paired-samples *t*-test (*df* = 25).

The mean and standard deviation of scores on the presence response scale were 0.28 (0.69), 0.25 (0.58), -0.10 (0.76) and -0.15 (0.56) for *PP*, *NP*, *PN* and *NN* respectively. A repeated-measures univariate ANOVA was conducted on participants’ score on the presence response scale to test the effect of bystanders’ attitude towards both virtual peer speakers and participants. The result showed a significant effect of bystanders’ attitude towards the participants *F*(1,25) = 7.21, *p* = .013, *η^2^* = .22 as participants rated their feeling of presence higher when bystanders had a positive instead of negative attitude towards them, see Fig 7. No additional significant effects were found when the analysis was repeated including only the male participants (Table C in S2 Table) or when including participants’ gender as a between-subject factor (Table C in S3 Table).

### 3.3 Speech length

**Fig 8:**
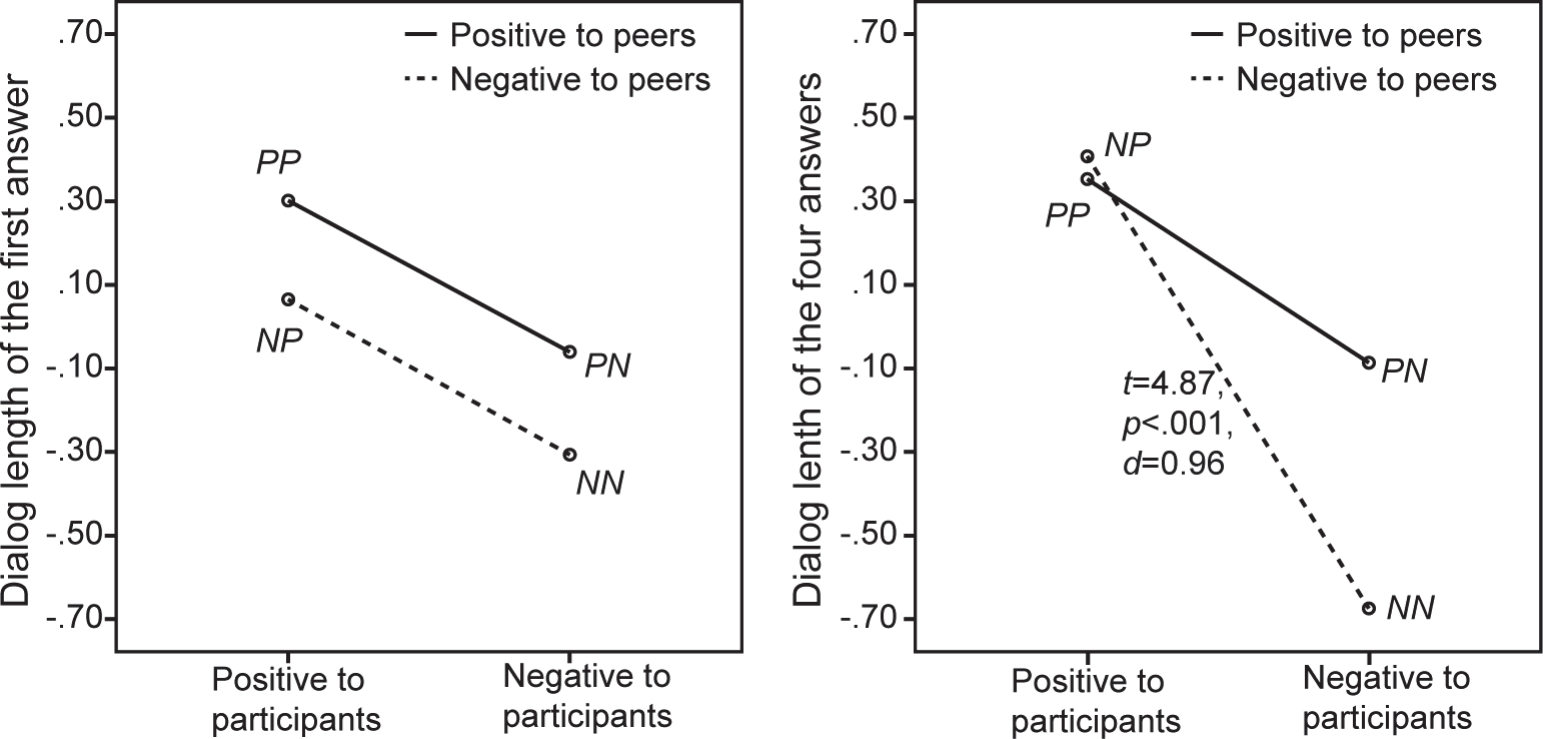
Participants’ dialogue length, including the result of a paired-samples *t*-test (*df* = 25).

Mean and standard deviation of participants’ total speech length were 0.35 (0.63), 0.41 (0.67), -0.09 (1.00) and -0.67 (0.70) for *PP*, *NP*, *PN* and *NN* respectively, as also shown in Fig 8. A repeated-measures univariate ANOVA was conducted with the same two within-subject factors as before on participants’ dialogue length in each session. The results showed a significant main effect for the bystanders’ attitude towards the participants, *F*(1,25) = 19.78, *p* <. 001 *η^2^* = .44. Participants gave longer answers when bystanders’ attitude was positive instead of negative towards them. No significant two-way interaction was found, *F*(1,25) = 2.67, *p* = .12, *η^2^* = .096.

The answer's length for the participants’ first question was analyzed to examine the effect of the within-subject factors at the start of a participant’s turn to speak. The means and standard deviations of the participants’ speech length on the first question were 0.30 (0.74), 0.07 (0.90), -0.06 (0.97) and -0.31 (0.79) in the *PP*, *NP*, *PN* and *NN* conditions respectively. A repeated-measures univariate ANOVA was conducted using the dialogue length of the first question the participants answered as dependent variable. The result showed that the main effect for the bystanders’ attitude towards the participants approached the significant level, *F*(1,25) = 3.23, *p* = .084, *η^2^* = .11, with the answer's length being longer in the positive attitude condition than in negative one. Neither a significant main effect of the bystanders’ attitude towards the virtual peer speakers (*F*(1,25) = 1.54, *p* = .23, *η^2^* = .058), nor a significant two-way interaction (*F*(1,25) = 0.001, *p* = .98, *η^2^* <. 001) were found for the speech length on the first question.

No additional significant effects were found when the analyses were repeated including only the male participants (Table C in S2 Table) or when including participants’ gender as a between-subject factor (Table C in S3 Table).

### 3.4 Physiological measurements

**Fig 9:**
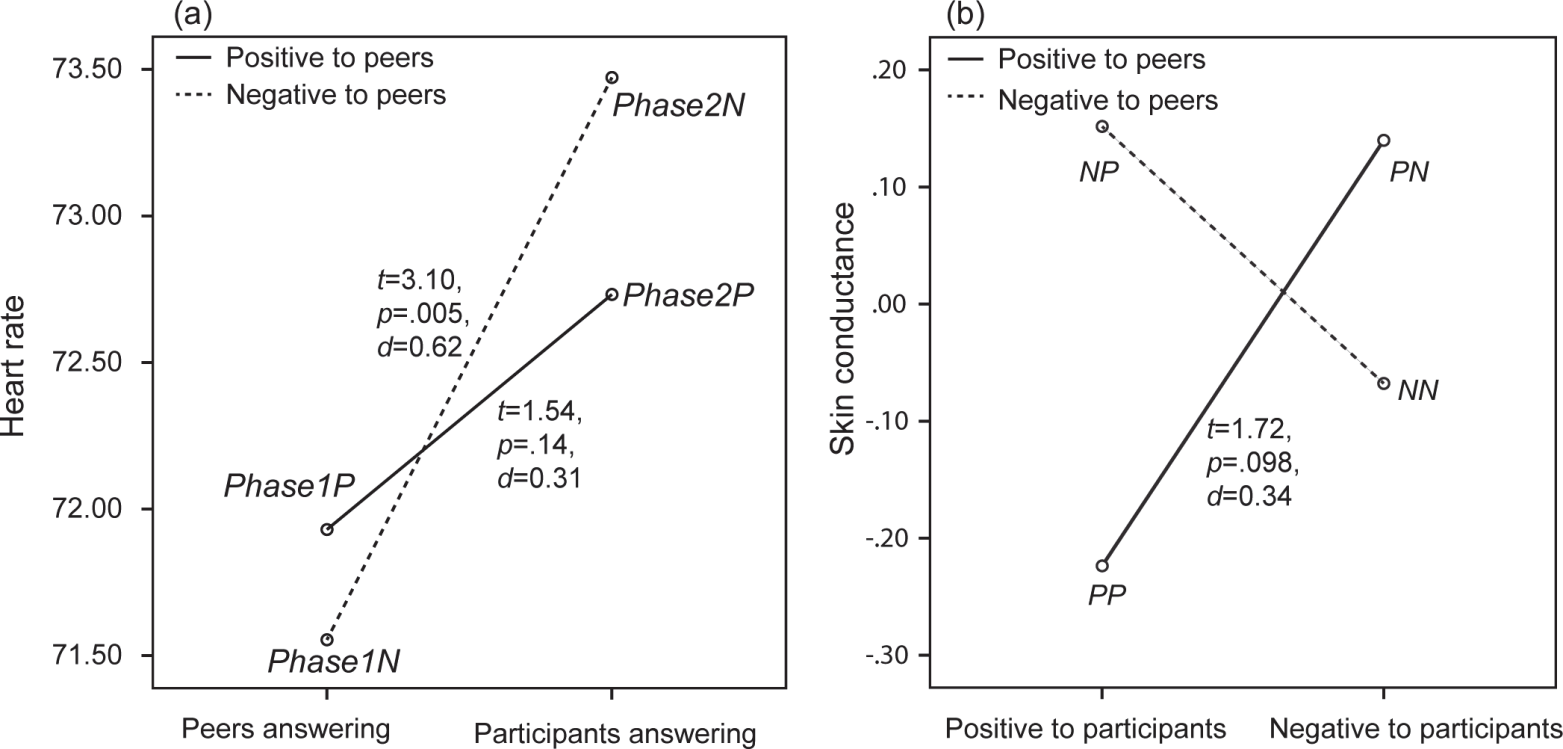
Participants’ heart rate when peers or participants were answering questions, including results of paired-samples *t*-tests (*df* = 24).

Means and standard deviations of heart rate and skin conductance are shown in Table 5. A repeated-measures univariate ANOVA was conducted using heart rate as dependent variable, and the phases of the lesson, the bystanders’ attitude towards virtual peer speakers and participants as independent variables. The results, given in Table 6, showed a significant effect for the phase on participants’ heart rate with an increase in participants’ heart rate in the second phase, where they answered questions. The analysis also found a two-way interaction effect between phase and bystanders’ attitude towards the virtual peer speakers, as also illustrated in Fig 9A. A significant increase in heart rate was found between the two phases only when in the first phase the participants observed a negative instead of positive attitude towards the virtual peers, *t*(24) = 3.10, *p* = .005, *d* = 0.63. If they first observed a positive attitude towards the virtual peers no significant difference was found between the two phases, *t*(24) = 1.54, *p* = .14, *d* = 0.31.

**Table 5:** Means and standard deviations of heart rate and skin conductance across the two phases of the four conditions.

	Phase 1—Peers answering				Phase 2—Participants answering			
	*PP*	*NP*	*PN*	*NN*	*PP*	*NP*	*PN*	*NN*
Heart rate	71.83	71.35	72.03	71.76	72.68	73.18	72.78	73.76
	(10.59)	(9.99)	(9.31)	(11.93)	(10.34)	(10.41)	(10.08)	(12.05)
Skin conductance	-0.29	0.12	0.11	-0.12	-0.16	0.18	0.17	-0.017
	(1.07)	(0.82)	(0.88)	(0.81)	(0.98)	(1.05)	(0.82)	(1.04)

**Table 6:** The results of repeated-measures univariate ANOVAs for heart rate and skin conductance.

Independent variables	Heart rate			Skin conductance		
	*F*(1,24)	*p*	*η^2^*	*F*(1,24)	*p*	*η^2^*
Phase	6.675	**.016**	**.22**	0.249	.62	.01
Attitude to participants	0.282	.60	.012	0.195	.66	.008
Attitude to peers	0.132	.72	.005	0.266	.66	.011
Phase × participants	0.003	.96	<.001	0.004	.95	<.001
Phase × peers	6.370	**.019**	**.21**	0.003	.96	<.001
Participants × peers	0.058	.81	.002	3.35	.08	.12
Phase × participants × peers	0.055	.82	.002	0.044	.84	.002

A similar repeated-measures univariate ANOVA was conducted with skin conductance as dependent variable. Although the results did not show any significant effect, Table 6 shows a two-way interaction between bystanders’ attitude towards the participants and virtual peer speakers approaching significance (*p* = .08). As Fig 9B shows when bystanders first expressed a positive attitude towards the virtual peer speakers, participants sweated more when after this bystanders expressed a negative attitude instead of a positive attitude towards them. Note again that this difference was only approaching an significant level (*t*(24) = 1.72, *p* = .098, *d* = 0.35).

No additional significant effects were found when the analyses were repeated including only the male participants (Table D in S2 Table) or when including participants’ gender as a between-subject factor (Table D in S3 Table).

### 3.5 Consistency

As the overall test failed to find a significant main effect for (in)consistency on changes in participants' beliefs (Table 2), post-hoc analyses were only conducted to find potential clues to modify the third hypothesis. The (*PP*-*NN*)^2^ = (*NP*-*PN*)^2^ contrast was examined for all the belief data collected. The results of paired-samples *t*-tests, given in Table 7, all failed to reach the Sidak corrected α level of. 0032 for 16 items. No significant effects were also found when the analysis was repeated including only the male participants (Table E in S2 Table) or when including participants’ gender as a between-subject factor (Table E in S3 Table).

**Table 7:** Means and standard deviations of consistent (*PP*-*NN*)^2^ and inconsistent (*NP*-*PN*)^2^ conditions, including the results of paired-samples *t*-tests (the Sidak corrected *α* = .0032).

Measurements	(*PP*-*NN*)^2^	(*NP*-*PN*)^2^	Paired-samples *t*-tests		
	Mean (*SD*)	Mean (*SD*)	*t*(25)	*p*	*d*
**The participants**					
P1 Own performance	0.26(0.32)	0.72(1.27)	-2.06	.05	-0.40
P2 Satisfaction with own performance	0.39(0.93)	0.97(1.25)	-2.00	.057	-0.39
P3 Other students’ satisfaction with your performance	5.43(3.85)	4.90(3.78)	0.70	.49	0.14
P4 Teacher’s satisfaction with your performance	0.43(0.59)	1.14(1.75)	-2.18	.04	-0.43
P5 Other students like you	5.22(4.13)	4.95(4.51)	0.28	.78	0.055
P6 Teacher likes you	0.66(0.91)	0.73(1.06)	0.29	.77	0.057
P7 Virtual lesson experience	1.44(2.10)	1.17(1.74)	0.60	.55	0.12
P8 Self-efficacy	0.40(0.80)	0.80(1.68)	-1.15	.26	-0.23
**Other students**					
S1 Other students’ performance	2.06(2.67)	1.03(1.22)	2.07	.049	0.41
S2 Participants’ satisfaction with other students’ performance	2.01(2.03)	1.02(1.19)	2.78	.01	0.55
S3 Participants like the other students	2.30(2.30)	1.63(2.08)	1.38	.18	0.27
S4 How supportive were the other students to you	7.29(4.79)	7.34(4.44)	-0.05	.96	-0.01
**The teacher**					
T1 The teacher’s performance	0.51(0.70)	0.51(0.87)	0.01	.99	0.002
T2 Participants’ satisfaction with teacher’s performance	0.82(1.35)	0.69(1.09)	0.41	.68	0.08
T3 Participants Like the teacher	0.76(1.41)	0.76(1.29)	0.01	.99	0.002
T4 How supportive was the teacher to you	0.96(1.72)	0.92(1.44)	0.09	.93	0.018

## Discussion and Conclusions

Given these results a number of conclusions can be drawn. First, virtual bystanders exhibiting positive instead of negative attitude towards the participants, make the participants to hold more positive beliefs about their own self-efficacy (supports *H1b*) and to behave more engaging by giving longer answers, i.e., showing less avoidance behavior which is interpreted as a manifestation of less anxiety (support *H1c*). Thus, these results confirm part of the hypothesis about the influence of bystanders’ attitude. Also, the two-way interaction on the self-reported anxiety showing that bystanders’ consistent positive attitude towards both the peer speakers and the participants evoked the lowest level of anxiety in the participants, supports *H1c*. Although no significant effect for bystanders’ attitude toward the participants on participants’ perceived performance (*H1a*) was found for the whole dataset, the effect approached significance in the hypothesized direction.

Second, as predicted the participants seem to have experienced anticipation anxiety as their heart rate increased when they had to actively answer the teacher’s question after passively observing bystanders exhibiting especially a negative attitude towards the virtual peer speakers (supports *H2b*). More support for the second hypothesis was found in the two-way interaction on the self-reported anxiety (*H2b*) and self-efficacy (*H2a*). For self-reported anxiety, it seems that any of the two types of negative attitude displayed increased anxiety. Still being negative at the same time towards both the virtual peer speakers and the participants did not seem to elicit more anxiety than the conditions where bystanders showed negative attitudes only towards the virtual peer speakers or the participants. For self-efficacy, it seems that once the participants had witnessed the bystanders’ positive attitude to their virtual predecessors, a positive attitude towards them gave their self-efficacy a boost while a negative attitude a blow.

A third conclusion that can be drawn relates to the effect of consistency and inconsistency between bystanders’ attitudes. The third hypothesis predicted that inconsistency would cause larger changes in participants’ beliefs than consistency. However, both the overall analysis and post-hoc analysis of the separate belief items failed to find a significant effect. This implies that no support or additional clues were found for this hypothesis.

Support for the fourth hypothesis about virtual flattering and destructive criticism was also found. When other students were flattering instead of criticizing destructively the participants, the participants rated the students’ performance higher, were more satisfied with their performance, liked them more, and found them more supportive. Interestingly, this effect also rubbed off to the neutral teacher as similar effects were also found for participants’ beliefs about the teacher. Still, instead of simply rubbing off, in the debriefing some participants mentioned that they regarded the neutral stance of the teacher as inappropriate, since they expected him to intervene when students were openly making negative comments.

Besides the predicted effects, the experiment also revealed some unexpected findings when it came to participants’ feelings of presence; participants rated their presence higher when the bystanders exhibited a positive instead of a negative attitude towards them. This again could be a case of rubbing off, i.e., towards the quality of the virtual reality environment. The cognitive dissonance theory [48], however, also offers an explanation. As participants experienced inconsistency between the bystanders’ negative attitude towards them and the positive self-image participants probably held, participants could have resolved this inconsistency by changing their belief about the credibility of the virtual experience; in other words, they could start questioning the plausibility, regarding it as dissimilar to their beliefs of the real world [60, 69].

Like any empirical study, this experiment also had a number of limitations that should be noted. First, the task in the virtual environment was quite familiar to the participants which might have limited the effect of vicarious experience as vicarious experiences are a particularly valuable source of reassurance mainly when people are unsure about their own capabilities [24, 70]. Future work could therefore test the vicarious experience in scenarios where individuals lack direct knowledge of their own capabilities, such as in a virtual teaching lesson or acting lesson, where they would rely more heavily on modeled indicators. Second, the participants were asked to answer questions more often than the other virtual students, which could have lowered their vicarious experience, but gave them more exposure to the bystanders’ direct evaluation. It would, however, also be interesting to study the effect of virtual bystanders in a more normal full-length English lesson of around 45 minutes where people spend more time observing others instead of speaking themselves. Third, to avoid interrupting the flow of the experience no self-reported data were collected directly after the participants witnessed their virtual students’ answers. However, these data would give insight into the effect of vicarious experience on self-efficacy and anticipation anxiety just before it was the participants’ turn to speak. Still, the collected heart rate data did provide some insight into their anxiety. However, since knowing the process of participants’ anxiety level during the exposure is valuable for therapists, future studies might consider measuring subjective unit of discomfort at different moments during the exposure as some studies have already successfully done [71, 72]. Fourth, neither the attitude of the bystanders nor the response of the teacher changed as a reaction towards the participants’ performance. Still others have shown that providing positive or negative feedback in a dialogue can affect people’s emotion and behavior [8, 71]. Therefore, making bystanders or the teacher adapt their attitude to the performance of participants might affect participants’ motivation as they could experience that their effort could have an impact on their environment. Fifth, the study showed that participants’ gender could affect their experience. As the sample in this experiment is male biased, generalization of the findings towards a female population should be done with caution. One potential explaining factor for a possible gender effect might have been the gender of the teacher, which was always male. Although the teacher was not considered as a bystander, but as the primary communication partner in this experiment, future work could explore also the effect of virtual bystanders when including a female teacher. Furthermore, this experiment used an opportunity sampling strategy, resulting in a sample with mainly male participants. Future work therefore might, possibly in combination with a female teacher, include more females in the sample, as this would provide more understanding into same-gender or mixed-gender interaction styles. Finally, this experiment only recruited university students as participants, and their PRCS data suggested that they were generally socially confident. No clinical measures or inclusion criteria were used to identify participants with possible mental disorders in the sample, but based on the PRCS data these additional measures and criteria would hardly have affected our group of participants. Yet, for further generalization of our findings, it would be interesting to study how virtual bystanders would affect other groups of people, such as patients that suffer from social anxiety disorder.

The main contribution of the research presented is to establish insight into the effect of virtual bystanders in a virtual reality environment. The attitude expressed by them can have a clear effect on people’s beliefs, self-efficacy, and anxiety. Therefore, manipulating the virtual bystanders’ attitude could give therapists a tool to control the exposure in virtual reality environments for the treatment of social anxiety disorder. Another contribution of this work is the insight it provides into classroom dynamics. The simulation in the virtual classroom suggests that fellow classmates exhibiting a positive attitude towards each other leads students to act more engaging, to have more self-efficacy and to experience less anxiety, while a negative attitude could have a detrimental effect on all these aspects. Although teachers might take a neutral stance towards the class attitude, it still forms students’ beliefs towards them. To conclude, the virtual bystander seems to have a clear ability to have an impact on the social experience in virtual environments that seem to correspond to what people experience in everyday life.

## Supporting Information

S1 Table The data of all the 26 participants. (XLSX)

S2 Table Hypotheses, the corresponding analyses and results using the dataset with male participants only. (XLSX)

S3 Table Hypotheses, corresponding data analyses (repeated-measures MANOVAs and ANOVAs) and results using bystanders’ attitude towards (1) the virtual peer speakers and (2) towards the participants as two independent within-group factors, gender as an independent between group factor and as dependent variables: BEQ, PRS, post SUD, pre-post SUD, speech length, heart rate and skin conductance. Note that phase was also included as an independent variable for heart rate and skin conductance for subsequent ANOVAs.
(XLSX)

S1 Text The belief and experience questionnaire (BEQ). (PDF)

S1 Video An example of the interaction in the virtual classroom. (MPEG)
