# Supplementary material for: Virtual Bystanders in a Language Lesson: Examining the Effect of Social Evaluation, Vicarious Experience, Cognitive Consistency and Praising on Students' Beliefs, Self-Efficacy and Anxiety in a Virtual Reality Environment
Source: PLoS One. 2015 Apr 17;10(4):e0125279. doi: 10.1371/journal.pone.0125279 (PMC4401547; doi:10.1371/journal.pone.0125279)
Supplement: S1 Text — (PDF) [file pone.0125279.s004.pdf]

Virtual bystanders in a language lesson: examining the effect of social evaluation, vicarious experience, cognitive consistency and praising on students' beliefs, self-efficacy and anxiety in a virtual reality environment

Chao Qu, Yun Ling, Ingrid Heynderickx, Willem-Paul Brinkman

Text S1: The belief and experience questionnaire (BEQ)

With regard to the English lesson in the last session:

P1: How would you rate your performance? 11-point scale from 0 (very bad) to 10 (very good)

P2: How satisfied are you with your performance? 11-point scale from 0 (highly unsatisfied) to 10 (highly satisfied)

P3: How satisfied do you think the other students were with your performance? 11-point scale from 0 (highly unsatisfied) to 10 (highly satisfied)

P4: How satisfied do you think the teacher was with your performance? 11-point scale from 0 (highly unsatisfied) to 10 (highly satisfied)

P5: How much do you think the other students like you? 11-point scale from 0 (not at all) to 10 (very much)

P6: How much do you think the teacher likes you? 11-point scale from 0 (not at all) to 10 (very much)

P8: After the last session, how confident and competent do you feel now in giving answers in an English lesson in real life? 11-point scale from 0 (not confident at all) to 10 (very confident)

S1: How would you rate the performance of the other students? 11-point scale from 0 (very bad) to 10 (very good)

S2: How satisfied are you with the performance of the other students? 11-point scale from 0 (highly unsatisfied) to 10 (highly satisfied)

S3: How much do you like the other students? 11-point scale from 0 (not at all) to 10 (very much)

S4: How supportive were the other students when you were giving answers? 11-point scale from 0 (very unsupportive) to 10 (very supportive)

T1: How would you rate the performance of the teacher? 11-point scale from 0 (very bad) to 10 (very good)

T2: How satisfied are you with the performance of the teacher? 11-point scale from 0 (highly unsatisfied) to 10 (highly satisfied)

T3: How much do you like the teacher? 11-point scale from 0 (not at all) to 10 (very much)

T4: How supportive was the teacher when you were giving answers? 11-point scale from 0 (very unsupportive) to 10 (very supportive)

P7: Please indicate how you experienced the English lesson in the last session.

I experienced the lesson as:

P7.1: 11-point scale from 0 (unpleasant) to 10 (pleasant)

P7.2: 11-point scale from 0 (not relaxed) to 10 (relaxed)

P7.3: 11-point scale from 0 (aggressive) to 10 (non-aggressive)

P7.4: 11-point scale from 0 (uncomfortable) to 10 (comfortable)

P7.5: 11-point scale from 0 (impolite) to 10 (polite)

P7.6: 11-point scale from 0 (exhausting) to 10 (energizing)
